# Use of oral contrast in 2024: primer for radiologists

**DOI:** 10.1007/s00261-024-04409-2

**Published:** 2024-07-02

**Authors:** Aaroh Patel, Neeraj Lalwani, Ania Kielar

**Affiliations:** 1 Virgina Commonwealth University School of Medicine, Richmond, VA USA; 2 Section of Abdominal Imaging, Virgina Commonwealth University School of Medicine, Richmond, VA USA; 3 Joint Department of Medical Imaging, Toronto, Canada

## Introduction

Oral contrast has been a critical component in computed tomography (CT) imaging of the abdomen and pelvis (AP) for many years [1]. However, modern imaging techniques have significantly enhanced resolution and clarity, reducing the reliance on oral contrast agents for differentiating anatomical structures and pathology. The use of oral contrast, particularly in the emergency department (ED), remains controversial due to concerns over cost, increased radiation exposure, and potentially prolonged throughput times without substantial impact on patient management [2]. While CT of the AP without oral contrast is commonly ordered for cases of acute abdominal pain, there are specific conditions where oral contrast may significantly improve diagnostic accuracy and patient care [2].

In this manuscript, we discuss and review the indications for the judicious use of oral contrast in CT of the AP as of 2024, considering the latest advancements and ongoing debates in clinical practice.

## Oral contrast: types and roles

Oral contrast can be broadly classified as positive and neutral. Positive oral contrast consists mainly of barium-sulfate suspensions or water-soluble iodinated solutions. These appear radiopaque on CT images, enhancing contrast resolution between bowel loops and surrounding structures, and facilitating the visualization of suspected inter-bowel loop abscesses, post-operative bowel anastomotic leaks, extraluminal soft tissue density tumors, and bowel fistulas [3].

### Barium-based vs. iodine-based oral contrast

- Barium-Based Oral Contrast: These agents are dense, radiopaque solutions that provide excellent mucosal coating. They are less likely to be absorbed systemically, making them safer for patients at risk of adverse reactions to iodinated contrast. However, they are contraindicated in patients with suspected bowel perforation due to the risk of barium peritonitis.
- Iodine-Based Oral Contrast: These agents are preferred in cases where there is a risk of bowel perforation because they are absorbed into the bloodstream and excreted by the kidneys, reducing the risk of severe inflammatory response if leakage occurs. However, iodine-based contrasts are more expensive and can be less palatable, affecting patient compliance.

In the case of post-operative bowel anastomotic leak evaluation, radiologists must ensure the water-soluble oral contrast has reached anastomosis. This may require the use of either oral or/and rectal contrast and may also require delayed imaging or repeat imaging with CT to ensure that enough time has passed for the oral contrast to reach and pass through the anastomosis.

The benefit of positive contrast has also been described in the literature for the assessment of non-acute or sub-acute nonspecific abdominal pain that is poorly localized. In this non-acute setting, a comprehensive examination with oral contrast may be used to identify subtle pathology as patient throughput time becomes less of a concern. However, there is no consensus on guidelines to support this practice. Several studies have identified oral contrast as non-contributory to radiologic diagnoses in the majority of patients presenting with acute non-traumatic abdominal pain [2]. On the other hand, the use of intravenous contrast remains uncontroversial and can impact the diagnostic accuracy of acute abdominal pain, with differences as significant as 30% in diagnostic accuracy compared to non-enhanced CT having been reported [4].

The use of positive oral contrast in cancer staging and peritoneal carcinomatosis surveillance has been documented in the literature [5]. Staging of tumors that involve the bowel or peritoneum may be facilitated with positive oral contrast to enhance the separation of the bowel from nearby masses. Furthermore, cancer staging is often conducted in a non-urgent outpatient setting, eliminating a key disadvantage of oral contrast use, namely turnaround time. Despite some isolated studies disputing the use of oral contrast in the evaluation of oncologic patients, CT of the AP with administration of oral contrast remains standard practice. Understanding key peritoneal or retroperitoneal pathways for the spread of various malignancies can help radiologists to better evaluate CT images even without oral contrast. Unfortunately, in cases where there is calcification of peritoneal metastases, including mucinous ovarian malignancies, the use of oral contrast may lead to false-negative reports of peritoneal disease [5]. Furthermore, advancements in modern CT scanners and image rendering provide increased resolution, which may diminish the benefits of oral contrast in cancer staging and peritoneal carcinomatosis.

Positive oral contrast may also be used for CT colonography, a procedure recommended by the U.S. Preventive Services Task Force as an alternative to optical colonoscopy due to its safety and efficiency. In CT colonography, oral contrast agents are used to tag fluid and stool; however, these agents also tag polyps, especially those of villous histology, in a distinctive pattern. Thus, oral contrast in combination with CT colonography may play a role in the recognition and screening of colorectal cancer [6].

Neutral oral contrasts include water or water-attenuated contrast agents such as mannitol and polyethylene glycol. These contrast agents aid in visualizing luminal content and the bowel wall. Neutral oral contrast is widely used when evaluating inflammatory bowel disease (IBD), intraluminal filling defects including neoplasms, causes of recurrent gastrointestinal (GI) bleeds, and bowel wall calcifications. However, neutral enteric contrasts struggle to visualize extraluminal soft-tissue density lesions and bowel fistulas [7]. Therefore, neutral enteric contrast is preferably used in CT enterography and some indications for CT angiography (CTA) due to the masking of mucosal enhancement and the alteration of three-dimensional (3D) volume-rendered vascular images with positive enteric contrast [8]. With CT enterography replacing small-bowel follow-through as the primary diagnostic modality for small-bowel imaging, neutral oral contrast serves as an essential component of the gold-standard imaging modality for the diagnosis and follow-up of Crohn’s disease.

## Oral contrast: contraindications and limitations

Positive enteric contrast should never be used in acute GI bleeds, as it obscures contrast-enhanced blood within the bowel lumen [9]. In addition, using positive oral contrast in diagnosing acute pain and bowel ischemia is not recommended due to the increased time needed to opacify the bowel with oral contrast and associated challenges in seeing bowel wall density and enhancement compared to the dense intraluminal oral contrast. Positive enteric contrast is also not recommended for the imaging of hepatobiliary, pancreatic, or genitourinary indications as it provides no direct diagnostic benefit [1].

Broadly speaking, positive oral contrast should never be used in CT enterography, suspected intra-abdominal hemorrhage or gastrointestinal bleeding, CTA, or blunt abdominal trauma (acute). In these cases, the use of positive oral contrast may obscure critical findings, interfere with diagnostic accuracy, or pose additional risks to the patient, highlighting the importance of careful consideration and adherence to established guidelines when selecting contrast agents for imaging studies.

Neutral enteric contrast is often preferred over positive oral contrast when assessing vessels. Positive oral contrast can negatively affect the evaluation of mucosal enhancement in cases of bowel wall thickening, mesenteric ischemia, enteritis, and angioedema. However, it does not typically impact the assessment of intramural edema, which is crucial for diagnosis. Positive contrast may alter the appearance of 3D vascular images but usually does not affect the two-dimensional (2D) source images crucial for diagnosis. While small bowel obstruction (SBO) is considered a contraindication by some, it has been found useful in practice to diagnose or rule out SBO and to provide a functional assessment [1]. Positive oral contrast in patients with suspected SBO helps in general assessment. Furthermore, for patients presenting with constipation due to opioid use or other causes, the water-soluble iodinated contrast material can have a therapeutic benefit.

An interesting connection between positive and neutral oral contrast is that one’s disadvantage is often the other’s advantage. For example, neutral oral contrast can detect causes of GI bleeds, a contraindication of positive oral contrast. On the other hand, positive oral contrast can help identify or characterize extraluminal masses and fluid (i.e., hematoma), while neutral contrast attenuates similarly to these conditions [5, 10].

Contraindications of oral contrast (positive or neutral) use are generally related to concerns of underlying conditions requiring the patient to be on volume restriction, allergies to contrast media (most commonly seen with intravenous contrast use, but a small portion of oral contrast is absorbed from the GI tract), and risk of aspiration [11]. Fortunately, some of these contraindications have accessible solutions, such as an enteric tube-mediated contrast administration for patients at risk for aspiration. For patients with known allergies to contrast media, the American College of Radiology (ACR) has laid down guidelines and recommends dilution of the contrast agent and premedication prophylaxis with steroids or antihistamines [11].

As mentioned before, oral contrast use should be reconsidered when throughput time is critical. According to several studies, the omission of oral contrast use in CT abdomen demonstrates a significantly decreased length of stay in the ER, reducing costs and improving patient satisfaction [12]. Summary of Studies on Throughput Time through ED: See Table 1 [13–15].

**Table 1:** Multiple studies showing significantly reduced ER length of stay in non-contrast-enhanced CT compared to routine oral contrast use

Author	Year	Number of patients	Reduced ER length of stay (min)
Razavi SA	2014	375	7
Hopkins CL	2012	174	0
Levenson	2012	1992	4
Kepner	2012	2668	1

Restriction of oral contrast use also eliminates potential side effects such as bloating, nausea, and cramping. However, low-osmolar contrast agents such as iohexol may confer better side-effect profiles because they lead to less endoluminal fluid shifts and less resultant nausea, diarrhea, and electrolyte abnormalities [12]. Furthermore, taste, while subjective, differs between contrast media with different contrast agents conferring small but statistically significant differences in the satisfaction levels of patients. Agents such as iohexol may be preferred over agents with worse taste profiles (diatrizoate meglumine) when the diagnostic accuracy remains the same [12].

In terms of radiation exposure, positive oral contrast and certain neutral contrasts lead to higher radiation exposure than when water is ingested [16]. For example, in patients with histologically diagnosed Crohn’s disease, low-dose abdominal imaging (CT) has shown higher radiation dosages with polyethylene glycol (PEG) (neutral contrast) than with 2% Gastrografin (positive contrast) across most body mass index (BMI) subgroups (107.60 ± 78.7 mGy.cm vs 85.65 ± 58.2 mGy.cm)[16]. The greatest difference in radiation dose between PEG and the positive contrast group was within the BMI < 25 subgroup, likely due to patients with high BMI behaving differently with other parameters such as automatic tube current modulation. While the mechanism of action for such findings is unclear, the greater osmotic effect of PEG likely results in larger intraluminal fluid volumes and consequently increased beam attenuation. While there is a clear relationship between higher BMI and increased radiation dose due to greater attenuation of X-rays, the addition of positive oral contrast may not significantly change the percentage of dose alteration for BMI. The actual impact on radiation dose may be minimal, as modern CT scanners and their automatic exposure control systems adjust the dose based on the attenuation seen on the scanogram, which includes the effects of both body size and any orally administered contrast [1].

The use of oral contrast in patients with low BMI, especially those presenting to the emergency department with abdominal pain, has been shown to be beneficial in certain studies. A multicenter study demonstrated that 83% of patients with a BMI of 21 or lower had inadequate intraabdominal and intrapelvic fat to effectively separate anatomical structures on CT images without oral contrast [17]. This makes oral contrast particularly valuable in improving diagnostic accuracy in this population. Using body fat percentage measurements via bioelectric impedance analysis alongside BMI can help identify patients who would benefit most from oral contrast, thereby enhancing imaging clarity and diagnostic outcomes. Thus, BMI and body-fat percentages may be considered algorithmically to streamline oral-contrast use in the ED.

Overall, measures to reduce oral contrast use may lead to improved patient satisfaction, decreased throughput times, and cost reductions, each a valuable measure of competency for healthcare systems. However, these upsides only remain advantageous if diagnostic accuracy is not reduced in pursuit of non-medical fiscal incentives.

Summary of Oral Contrast Findings: See Table 2.

**Table 2:** Summary of various oral contrasts

Type of oral contrast	Indications	Not Indicated
Positive oral contrast	- Suspected inter-bowel loop abscesses - Post-operative bowel leaks* - Extra-luminal soft tissue density tumors - Cancer staging and peritoneal carcinomatosis surveillance - Bowel fistulas* - Non-acute, nonspecific abdominal pain (in certain contexts) - Evaluation of small bowel obstruction* - CT colonography for colorectal cancer screening	- Acute gastrointestinal bleeds - Diagnosing acute abdominal pain (due to time needed for opacification and visual obstruction) - Imaging of hepatobiliary, pancreatic, or genitourinary areas - CT enetrography - Suspected intraabdominal hemorrhage or gastrointestinal bleeding - CT angiography- Blunt abdominal trauma (acute)
Neutral oral contrast	- Evaluating inflammatory bowel disease (IBD) - Intraluminal filling defects including neoplasms - Causes of recurrent GI bleeds - Bowel wall calcifications - CT enetrography (replacing small-bowel follow-through for small-bowel imaging)	- Instances where positive contrast is indicated due to its ability to better delineate certain structures and pathologies
Oral contrast (general)	- Certain specific diagnostic and therapeutic applications as mentioned in the indication’s columns for positive and neutral contrasts	- Underlying heart failure - Allergies to contrast media - Risk of aspiration - Situations requiring rapid throughput to avoid delays and increased costs - Use in patients with specific conditions where the contrast could obscure critical findings or where it provides no direct diagnostic benefit

## Consideration of the ACR appropriateness criteria as guidelines for clinicians

The debate surrounding the judicious use of oral contrast in CT abdomen imaging has sparked considerable discussion in the literature. While several studies have argued against its routine use in certain clinical scenarios, it is crucial to recognize the value of evidence-based guidelines provided by organizations such as the ACR. These guidelines outline disease-specific indications for oral contrast use, ensuring optimal diagnostic outcomes while minimizing patient discomfort and radiation exposure.

Adherence to the ACR appropriateness criteria may lead to improved patient outcomes and resource utilization, such as reduced length of stay and fewer unnecessary imaging studies when following ACR guidelines for small bowel obstruction evaluation [18]. By sharing such examples, clinicians can gain insights into the practical benefits of guideline adherence and feel more confident in implementing these recommendations in their practices.

Interdisciplinary collaboration between radiologists, gastroenterologists, and other healthcare providers is essential for effectively applying the ACR guidelines in clinical practice. By working together to review and interpret imaging findings, healthcare teams can ensure comprehensive patient care and optimal outcomes. Encouraging open communication and shared decision-making can help overcome barriers to guideline implementation and foster a culture of evidence-based practice.

## Variability in oral contrast use

Despite the ACR appropriateness criteria’s reputation as evidence-based, peer-reviewed guidelines, they are not universally accepted in clinical practice. A comprehensive literature review found limited mention of the ACR appropriateness criteria in the recently published literature, suggesting a corresponding low prevalence in clinical practice. The article suggests that a “lack of formal training” in the use of the ACR guidelines and other imaging order practices in medical education perpetuates a low rate of incorporation of these criteria into clinical practice [19]. Currently, decisions regarding oral contrast use may vary by institution and according to physicians’ personal preferences, contributing to inconsistency in diagnostic decisions. Even oral volume preparation and protocol may vary between institutions.

A significant number of institutions have moved to water-soluble iodinated contrast. The concentration of iodine in water-soluble contrast media can vary widely, ranging from 4 to 48 mg I/ml, depending on the desired opacity and the section of the bowel being visualized.

For optimal bowel opacification in CT, a solution containing 13 to 15 mg I/ml of iodine is generally recommended for oral (and rectal) administration in adults [11]. This concentration strikes a balance, providing adequate lower Hounsfield unit opacity in the proximal bowel while ensuring higher Hounsfield unit opacity in the distal bowel. This balance is necessary because dilute, hypotonic contrast solutions tend to become more concentrated as they pass through the bowel, potentially affecting the uniformity of the imaging. Institutions may create their own solutions based on this description. For example, 35 ml of commercially available iopamidol 370 (370 mg organically bound iodine per ml of solution), when diluted in 900 ml of water (total volume 930 ml), will provide approximately 13.9 mg Iodine/ml. The oral contrast can be administered 1 h prior to the CT exam, ensuring that the contrast medium has sufficient time to travel through the gastrointestinal tract, providing clear and comprehensive imaging results.

Development of shorter and more time-efficient protocols with equivalent efficacy and safety may increase acceptance of oral contrast in the ED setting. Standardized protocols among all institutions may also improve safety and reliability. With the ACR criteria being online and freely accessible for public use, greater emphasis must be placed on educating current and future physicians on the practicality and convenience of these criteria in guiding diagnostic decisions. In summary, the absence of universally accepted guidelines for oral contrast use introduces challenges to patient safety, consistency, and resource allocation, creating incentives to further study the efficacy of oral contrast use in CT of the abdomen to address these issues and enhance the overall quality of patient care.

## Future directions: photon-counting CT (PCCT) and dual-energy CT (DECT)

Photon-counting CT (PCCT) and dual-energy CT (DECT) represent significant advancements in imaging technology. Photon-counting CT, in particular has introduced improvements such as increased spatial resolution, enhanced soft-tissue contrast, reduced radiation exposure, and optimized use of contrast agents.

Regarding the application of oral contrast in PCCT and DECT, detailed guidelines are less prevalent. Nevertheless, the superior image quality and reduced noise offered by PCCT could allow for the use of smaller amounts of contrast agents while still achieving high-quality images [20]. The ability of PCCT to operate in spectral modes—differentiating X-ray photons by their energy—enhances the optimization of contrast usage, potentially enabling more precise tissue characterization [21].

In the DECT context, the technology’s capability to employ two distinct X-ray photon energy spectra improves material characterization, including that of contrast agents, providing unique opportunities for contrast optimization.

The superior image quality produced by these novel imaging modalities is further enhanced by advancements in image rendering and display. Three-dimensionally rendered images combine axial sections to generate a comprehensive image that facilitates the discernment of anatomical relationships not visualized in the axial sections alone [22]. In the past, creating 3D-rendered images was impeded by demanding computational requirements and lengthy processing times; however, improvements in computational power have led to the acceptance of 3D images in clinical practice. Today, these images are mainly constructed using the volume rendering technique; however, novel algorithms such as cinematic rendering may offer further improvements in areas such as depth perception and soft-tissue visualization. Cinematic rendering with positive oral contrast administration leads to photorealistic depictions of bowel mucosal fold patterns that demonstrate bowel anatomy and pathology without artifacts. The contrast-opacified bowel synergizes well with the high levels of detail and shadowing provided by cinematic rendering, providing quality images that liken the technique to a form of “virtual fluoroscopy”[23]. Combining PCCT/DECT, oral contrast, and 3D rendering offers clinicians a new approach to exploring anatomic features with greater clarity and depth, facilitating the visualization and interpretation of various clinical conditions. This combination may also impact fields other than radiology, including medical education in anatomy, general patient education, and surgical planning [22].

Focused on this interplay, a growing area of research focuses on creating contrast agents that combine the strengths of positive and neutral contrast. Novel agents such as dark oral contrast and high-Z oral contrast may be examples of this [24]. These agents are currently undergoing clinical trials and development; in the future, they may provide higher levels of attenuation and clarity in combination with advancing imaging techniques. Dark oral contrast agents, more radiolucent than water but less so than gas, offer excellent bowel wall delineation and reduce imaging ambiguities. These agents, including paraffin and corn oil emulsions and perfluorocarbon-based liquids, have been in development since the 1970s and are currently under clinical consideration. They demonstrate potential in improving bowel imaging by offering a distinct attenuation profile on CT, beneficial for reducing motion artifacts and enhancing contrast in multi-energy CT scans.

High-Z element contrast agents achieve superior material separation in dual-energy CT. Agents based on elements such as tungsten, tantalum, and rhenium can be distinguished from iodine, enabling more precise imaging. Photon-counting CT, in particular, facilitates even better differentiation of these agents due to the unique attenuation properties near their K-edge, allowing for the imaging of multiple high-Z contrast agents simultaneously without interference.

However, potential issues may arise with multi-energy CT, such as pseudo lesions and artifacts, but the benefits, including reduced artifacts and enhanced material separation, are also significant. The synergy of using multiple oral and intravascular contrast agents could revolutionize CT imaging, enabling “multi-color” imaging that could improve diagnostic accuracy and reduce interpretation times through enhanced visualization and AI integration.

The evolution of oral contrast agents—from traditional positive and neutral agents to innovative dark and high-Z element agents—promises to significantly improve CT imaging’s ability to delineate bowel and peritoneal diseases. These advances may lead to better detection and monitoring of various pathologies with reduced risks of masking critical findings and pave the way for more comprehensive and less ambiguous imaging techniques.

A separate avenue for advancing the use of oral contrast involves the development of novel drinking protocols that lessen small but pertinent deterrents to the use of oral contrast, such as taste profiles. A flavored beverage (Breeza; Beekley Medical, Bristol, Conn.) containing thickening agents (sorbitol, mannitol, and xanthan gum) was compared to the most common commercially available agent for enterography protocols, a low-Hounsfield barium suspension. The flavored beverage offers similar side-effect profiles but scored significantly higher in taste and patients’ willingness to repeat the drinking protocol [25]. As previously mentioned, while subjective, taste confers statistically significant differences in patient satisfaction between contrast media. Thus, further research into flavored beverages and suspensions may increase adherence to drinking protocols and thus improve both the consistency and quality of radiologic examinations.

However, the addition of new contrast agents adds a layer of complexity, necessitating interdisciplinary collaboration between gastroenterologists, radiologists, and surgeons, as well as further education on the topic to ensure the judicious use of the correct type of enteric contrast in clinical situations. Given the novelty of these technologies and the ongoing nature of research into their applications, consulting the latest research and recommendations from professional bodies such as the ACR is advisable. Clinicians utilizing these advanced CT forms and novel contrast media should remain abreast of the literature and any guidance issued by the manufacturers of their institutions’ CT systems.

**Fig. 1:**
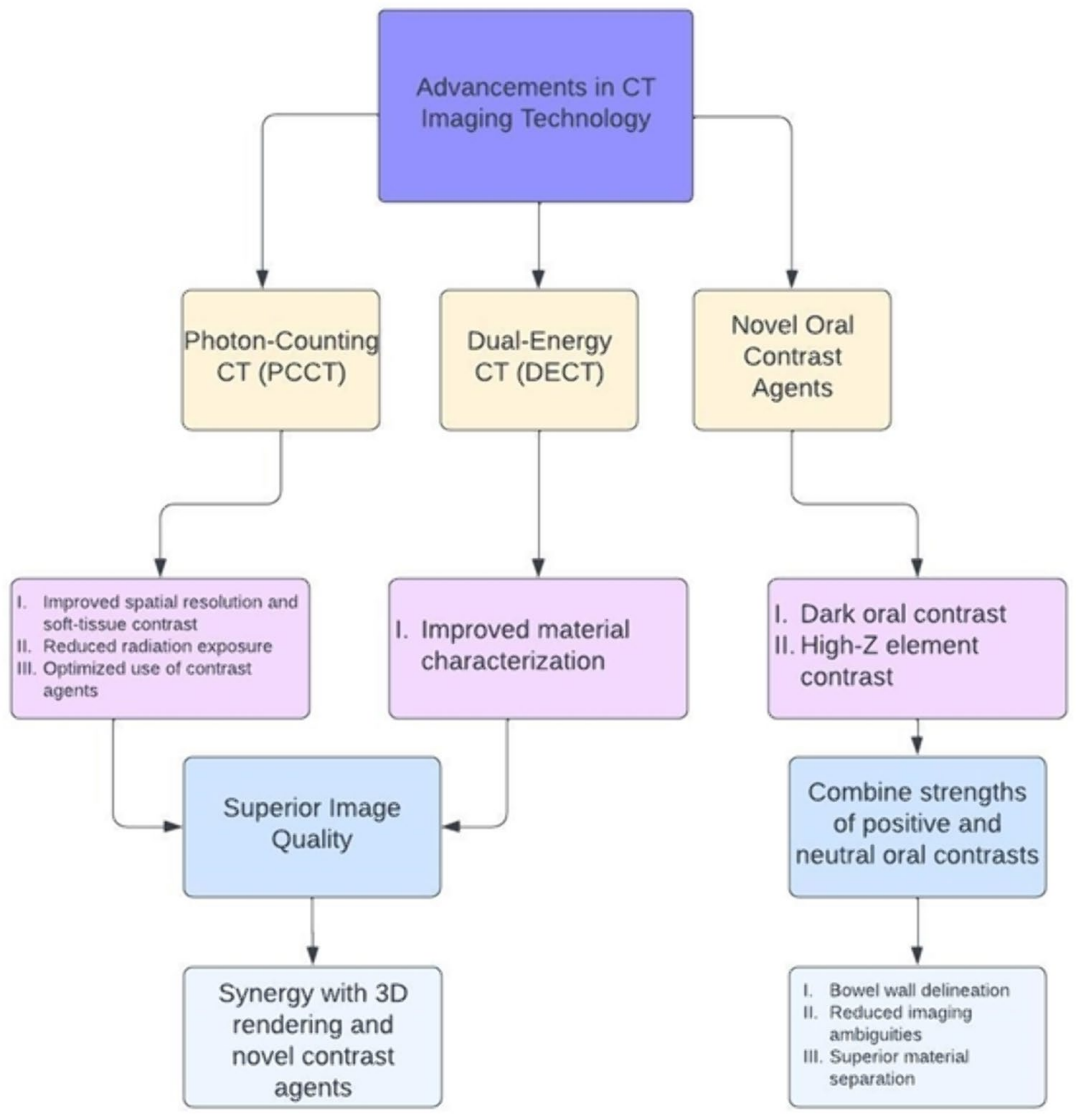
Summary of advancements in CT imaging technology

Summary of the Future Directions: See Fig. 1

## Conclusion

The use of oral contrast media in CT imaging of the abdomen and pelvis continues to be a topic of debate in medical centers worldwide. The benefits of positive oral contrast in specific conditions, such as cancer staging and postoperative assessments, have been highlighted, along with the advantages of neutral oral contrast in inflammatory bowel disease and CT enterography.

Despite advancements in imaging technology that have reduced the reliance on oral contrast, there are still clinical scenarios where its judicious use is critical. The development of standardized protocols for oral contrast administration, as well as ongoing research into novel contrast agents and imaging techniques, is essential for optimizing patient outcomes. Adherence to evidence-based guidelines, such as those provided by the ACR, can improve diagnostic accuracy, patient satisfaction, and resource utilization.

In summary, while the use of oral contrast in CT imaging remains contentious, it is clear that its strategic application can significantly enhance diagnostic precision in select cases. Future research and the establishment of universal guidelines will be critical in resolving existing controversies and advancing the field of radiologic imaging.
